# The nematode serotonin-gated chloride channel MOD-1: A novel target for anthelmintic therapy

**DOI:** 10.1016/j.jbc.2022.102356

**Published:** 2022-08-09

**Authors:** Noelia Rodriguez Araujo, Guillermina Hernando, Jeremías Corradi, Cecilia Bouzat

**Affiliations:** Instituto de Investigaciones Bioquímicas de Bahía Blanca, Departamento de Biología, Bioquímica y Farmacia, Universidad Nacional del Sur (UNS)-Consejo Nacional de Investigaciones Científicas y Técnicas (CONICET), Bahía Blanca, Argentina

**Keywords:** *C. elegans*, patch-clamp, Cys-loop receptors, MOD-1, 5-HT_3_, serotonin, anthelmintic drugs, 2-Me-5HT, 2-metyl-5-hydroxytryptamine, 5-HT_3_, serotonin type 3 receptor, ACh, acetylcholine, ECS, extracellular solution, EC_50_, half maximal effective concentration, GluCl, glutamate-gated chloride channel, GABA_A_, γ-Aminobutyric acid type A receptor, IC_50_, half maximal inhibitory concentration, IVM, ivermectin, Tryp, tryptamine, thy, thymol, LEV, levamisole, PZE, piperazine

## Abstract

Anthelmintics are used to treat human and veterinary parasitic diseases and to reduce crop and livestock production loss associated with parasitosis. The free-living nematode *Caenorhabditis elegans*, a model system for anthelmintic drug discovery, has a serotonin (5-HT)-gated chloride channel, MOD-1, which belongs to the Cys-loop receptor family and modulates locomotory and behavioral functions. Since MOD-1 is unique to nematodes, it is emerging as an attractive anthelmintic drug target, but details of MOD-1 function are unclear. Here, we revealed novel aspects of MOD-1 function from the molecular level to the organism level and identified compounds targeting this receptor, which may provide new directions for anthelmintic drug discovery. We used whole-cell current recordings from heterologously expressed MOD-1 to show that tryptamine (Tryp), a weak partial agonist of vertebrate serotonin type 3 (5-HT_3_) receptors, efficaciously activates MOD-1. A screen for modulators revealed that GABAergic ligands piperazine (PZE) and muscimol reduce 5-HT-elicited currents, thus identifying novel MOD-1 allosteric inhibitors. Next, we performed locomotor activity assays, and we found 5-HT and Tryp rapidly decrease worm motility, which is reversible only at low 5-HT concentrations. Mutants lacking MOD-1 are partially resistant to both drugs, demonstrating its role in locomotion. Acting as an antagonist of MOD-1, we showed PZE reduces the locomotor effects of exogenous 5-HT. Therefore, Tryp- and PZE-derived compounds, acting at MOD-1 through different molecular mechanisms, emerge as promising anthelmintic agents. This study enhances our knowledge of the function and drug selectivity of Cys-loop receptors and postulates MOD-1 as a potential target for anthelmintic therapy.

Parasitic nematodes have a great negative impact on human health. A great proportion of the world’s population, particularly those of underdeveloped and developing countries, harbors infections with helminths, which constitute the most common worldwide neglected tropical diseases. Parasitic nematodes also infect various types of organisms, including insects, animals, and plants and are therefore highly deleterious to veterinary health and agriculture productivity. Chemotherapy is required for eliminating morbidity associated with human and animal helminthiasis as well as for reducing crop and animal production loss. However, the emergence of nematodes resistant to the limited available drugs has become a global concern for veterinary and human health. Thus, new drugs and/or novel drug targets are urgently needed.

Nematodes are thread-like roundworms that can be parasitic or live in a wide range of environments including soil. The free-living nematode *Caenorhabditis elegans* shares physiological and pharmacological features with parasitic nematodes, including neurotransmitter receptors, and it is sensitive to most anthelmintic drugs (1, 2). Thus, by overcoming the disadvantages of working with parasites, *C. elegans* has been used as a valuable model for anthelmintic drug discovery (3).

Cys-loop receptors are major targets of anthelmintic drugs in parasites and *C. elegans* (2, 4). They belong to the family of pentameric ligand-gated ion channels that play key roles throughout the nervous system in vertebrates and invertebrates.

In vertebrates, Cys-loop receptors can be cationic channels, which include nicotinic acetylcholine receptor and serotonin type 3 receptors (5-HT_3_), or anionic channels, which include GABA (γ-aminobutyric acid) and glycine receptors. The repertoire in invertebrates is markedly broader and comprises also anionic channels activated by glutamate, acetylcholine (ACh), or serotonin (5-HT). The lack of these receptors in vertebrates make them attractive drug targets for antiparasitic therapies. Indeed, the glutamate-activated chloride channel (GluCl) is the target of the widely used anthelmintic drug ivermectin (IVM) (5). However, nematode resistance because of its extended and massive use has become a problem for animal and human health.

*C. elegans* possess a wide variety of serotonin receptors, including G protein-coupled receptors (SER-1, SER-4, SER-5, and SER-7) and a Cys-loop receptor named MOD-1, which is a chloride ion channel that mediates rapid hyperpolarizing responses in neurons and muscle (6, 7, 8, 9, 10, 11). A serotonin-gated cation channel, LGC-50, has been recently identified but does not show overlap in neuronal expression with MOD-1, suggesting they have distinct and separate roles in serotonergic signaling (12).

While MOD-1 and 5-HT_3_ share the ability to respond to 5-HT, they differ in their function in that 5-HT_3_ is a nonselective cation channel, whereas MOD-1 is a chloride channel not found in vertebrates (13). As an anionic Cys-loop receptor, MOD-1 shows about 30% identity with vertebrate GABA and glycine receptors.

MOD-1 expresses in *C. elegans* neurons and muscles that control behaviors, such as locomotion, egg laying, feeding, pharyngeal pumping, decision making, and aversive learning (14, 15, 16). It is also present in vertebrate parasitic nematodes, and it has been shown that *Haemonchus contortus* MOD-1 shows 80% identity with *C. elegans* MOD-1 (17). A search of transcriptome data for the filarial nematode parasite *Loa loa*, *Brugia malayi*, and *Wucheria bancrofti* revealed predicted coding sequences for orthologs of *mod-1* gene (17). MOD-1 is also present in plant parasitic nematodes, in which serotonergic signaling is important for plant invasion (18).

5-HT inhibits locomotion in *C. elegans* when applied exogenously, an effect that has been proposed to be mediated by both SER-4 and MOD-1 (19, 20). The 5-HT-induced paralysis differs from the classical spastic or flaccid paralysis and has been suggested to result mainly from the inability of worms to effectively integrate conflicting sensory inputs to initiate and sustain forward/backward locomotion (20). MOD-1 has been proposed as a potential drug target to control plant parasitic nematodes. In the potato cyst nematode *Globodera pallida*, MOD-1 participates in a serotonin signaling pathway that is essential for activation of the stylet that is used to access the host root (18).

Thus, ligands acting at MOD-1 can be attractive anthelmintic drugs expected to show secure toxicological profiles due to high selectivity for nematodes. However, little is known about MOD-1 molecular pharmacology and function.

Considering that MOD-1 can become an important novel anthelmintic target for parasites of humans, animals, and crops, we here revealed from the molecular level to the organism level novel aspects of its function and drug selectivity, and we identified MOD-1 ligands that may provide attractive scaffolds for anthelmintic discovery.

## Results

### Activation and desensitization properties of MOD-1

**Figure 1:**
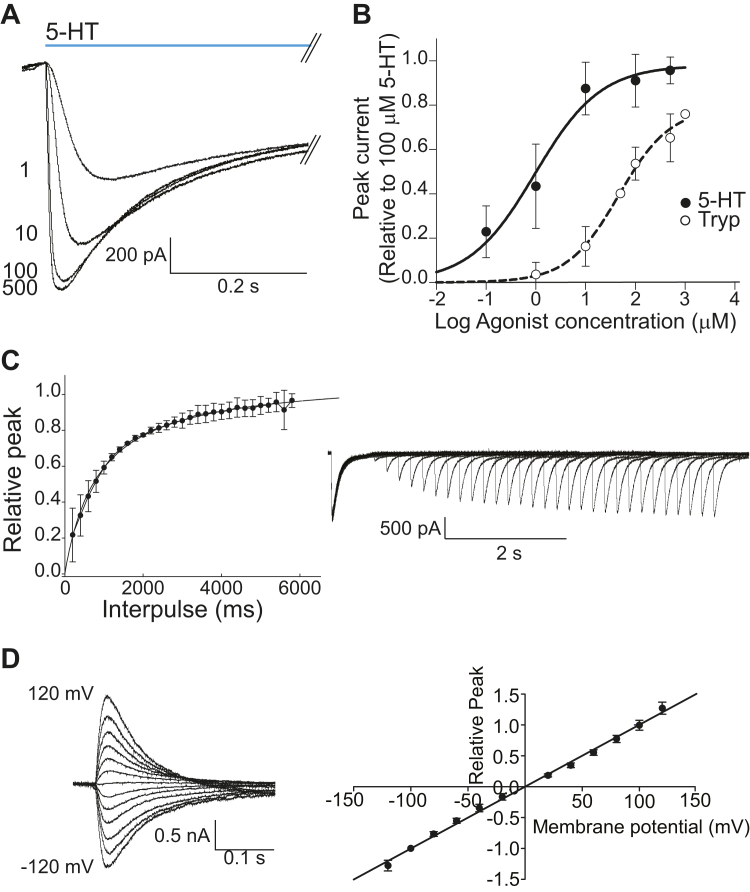
**Macroscopic characterization of activation of MOD-1**. *A*, typical whole-cell currents elicited by rapid application of 5-HT at the indicated concentrations (μM). Holding potential: −50 mV. Each current corresponds to the average current of three applications of 5-HT at a given concentration to the same cell. *B*, concentration–response curves for 5-HT and Tryp. The values correspond to the peak current relative to that elicited by 100 μM 5-HT in the same cell. Each point is shown as mean ± SD (n = 3). *C*, recovery of desensitization as a function of time after 5-HT removal. *Left*: Each point corresponds to the peak current elicited in the second 5-HT pulse relative to that elicited by the first agonist pulse. (n = 4). *Right*: Typical experiment showing the recovery of the original peak current as a function of time in the absence of 5-HT. Holding potential: -50 mV. *D*, current–voltage dependence of MOD-1. *Left*: Typical whole-cell currents elicited by 1 μM 5-HT at different holding potentials. *Right*: Current–voltage relationships obtained from the values of the peak current relative to that elicited at −100 mV holding potential for each cell. The points correspond to the mean ± SD (n = 3). The data were fitted by linear regression. 5-HT, serotonin; Tryp, tryptamine.

To decipher activation and desensitization properties of MOD-1, we expressed it in BOSC-23 cells and measured macroscopic currents elicited by rapid application of 5-HT at -50 mV holding potential (Fig. 1*A*). We constructed concentration–response curves by measuring at different agonist concentrations the peak currents, which were normalized to that elicited by a saturating 5-HT concentration (100 μM) in the same cell (Fig. 1, *A* and *B*). The resulting EC_50_ (half maximal effective concentration) for 5-HT was 0.96 ± 0.40 μM, which is in the same order as that of human and mouse 5-HT_3_A receptors (21).

At a saturating 5-HT concentration (100 μM), the rise time, measured by t_10-90%_ was 12.4 ± 4.1 ms (n = 10), which is similar to that of mouse and human 5-HT_3_A receptors (22). Decays were fitted by two exponential components, with time constants of 132 ± 1 ms (area 0.70 ± 0.09) and 1067 ± 290 ms (area 0.30 ± 0.15) (n = 5).

We measured the time course of recovery from desensitization using a twin pulse procedure (Fig. 1*C*). After a 3.5 s 5-HT pulse (100 μM) that allowed full desensitization, a pulse of free-agonist extracellular solution (ECS) was applied for variable durations in intervals of 200 ms, and then, a second pulse of agonist was applied. Fractional recovery was determined as the ratio between the peak current elicited by the second pulse of 5-HT respect to the first one for each cell. The plot of the percentage of recovery against the duration of the agonist-free interpulse was fitted by a single exponential with a time constant for recovery of 1.10 ± 0.007 s (n = 4), which is slightly faster than that of mouse 5-HT_3_A receptors (⁓4 s, (23)) (Fig. 1*C*).

To further characterize MOD-1 activation, we constructed current–voltage relationships (I/V) by measuring the peak current elicited by 1 μM 5-HT as a function of the holding potential (Fig. 1*D*). The reversal potential determined from the linear fit was −0.05 mV, which was similar to the theoretical value of the equilibrium potential for chloride under the present conditions (−3.4 mV). The magnitude of MOD-1 currents increased linearly with the voltage at both positive and negative holding potentials, indicating an ohmic behavior and little rectification.

### Searching for activators of MOD-1

**Figure 2:**
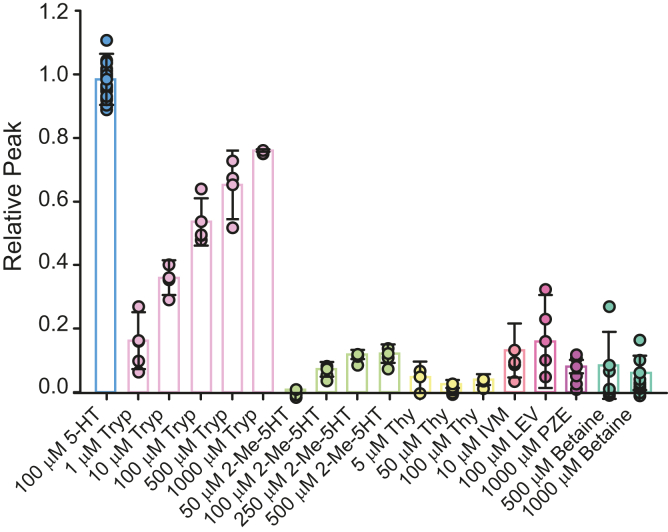
**Screening for MOD-1 activators**. Plot showing the maximal peak currents elicited by different ligands with respect to those elicited by a saturating (100 μM) concentration of 5-HT. Tryp: tryptamine, 1 μM (n = 4), 10 μM (n = 3), 100 μM (n = 4), 500 μM (n = 3), 1000 μM (n = 3); 2-Me-5HT: 2-metyl-5-hydroxytryptamine, 50 μM (n = 3), 100 μM (n = 3), 250 μM (n = 6), 500 μM (n = 6) Thy: thymol, 5 μM (n = 3), 50 μM (n = 6), 100 μM (n = 3); IVM: ivermectin, 10 μM (n = 5); LEV: levamisole, 100 μM (n = 4); PZE: piperazine, 1000 μM (n = 5); betaine, 500 μM (n = 6), 1000 μM (n = 9). The individual data points for each condition are shown with the colored bar indicating the mean ± SD. 5-HT, serotonin.

We first explored if agonists of vertebrate 5-HT_3_ receptors activate MOD-1. Tryptamine (Tryp) is a low-efficacy orthosteric agonist of mouse and human 5-HT_3_A receptors (21, 22). The concentration–response curve for Tryp revealed an EC_50_ value of 47 ± 11 μM and an efficacy (α) ⁓80% (Figs. 1*B* and 2). This result indicates that Tryp is markedly more efficacious for MOD-1 than for human (EC_50_ = 190 μM and α = 27%) and mouse (EC_50_ = 40 μM and α = 22%) 5-HT_3_A receptors (21, 22). We also evaluated the action of 2-Me-5HT (2-metyl-5-hydroxytryptamine), which elicits partial responses in mouse 5-HT_3_A receptors (EC_50_ = 5.2 μM and α = 60%; (21)). Application of 50 μM 2-Me-5HT (n = 3) did not show detectable responses. Increasing the concentration to 100 μM (n = 3), 250 μM (n = 6), and 500 μM (n = 6) resulted in very small responses, indicating that 2-Me-5HT has an extremely low ability to activate MOD-1 (Fig. 2). Thus, although MOD-1 and 5-HT_3_A show similar sensitivity for 5-HT, they respond differently to other orthosteric 5-HT_3_ agonists.

Terpenoids, such as thymol (Thy), are allosteric agonists of 5-HT_3_A receptors; they elicit activation by binding to a transmembrane cavity in the receptor (22, 24). We found that Thy at concentrations at which it is active at 5-HT_3_A receptors did not produce any significant response [5 μM (n = 3), 50 μM (n = 6), 100 μM (n = 3)], thus indicating that it is not an allosteric agonist of MOD-1 (Fig. 2).

We also tested if MOD-1 can be activated by the classical anthelmintic drugs, IVM, levamisole (LEV), and piperazine (PZE), that are agonists of GluCl, L-AChR (LEV-sensitive acetylcholine receptor), and UNC-49 receptors, respectively (5, 25, 26, 27, 28), as well as by betaine, which activates *C. elegans* ACR-23 and DEG-3/DES-2 receptors and possesses nematocidal activity (29, 30, 31, 32, 33). We used concentrations that have been applied for activation of their canonical target receptors. For 10 μM IVM (n = 5), 100 μM LEV (n = 4), 1 mM PZE (n = 5), 500 and 1000 μM betaine (n = 6 and n = 9) responses were negligible, indicating that these drugs do not activate MOD-1 (Fig. 2). In the case of IVM, we also verified that MOD-1 currents were not elicited if the drug was applied during 2 min (10 μM IVM; n = 5) as it has been shown that it produces slow activation of the GluCl receptor (34).

### Drug modulation of MOD-1 receptors

To contribute to the understanding of the role of this receptor in worms and to the development of novel anthelmintic drugs, we searched for compounds acting as modulators of MOD-1.

**Figure 3:**
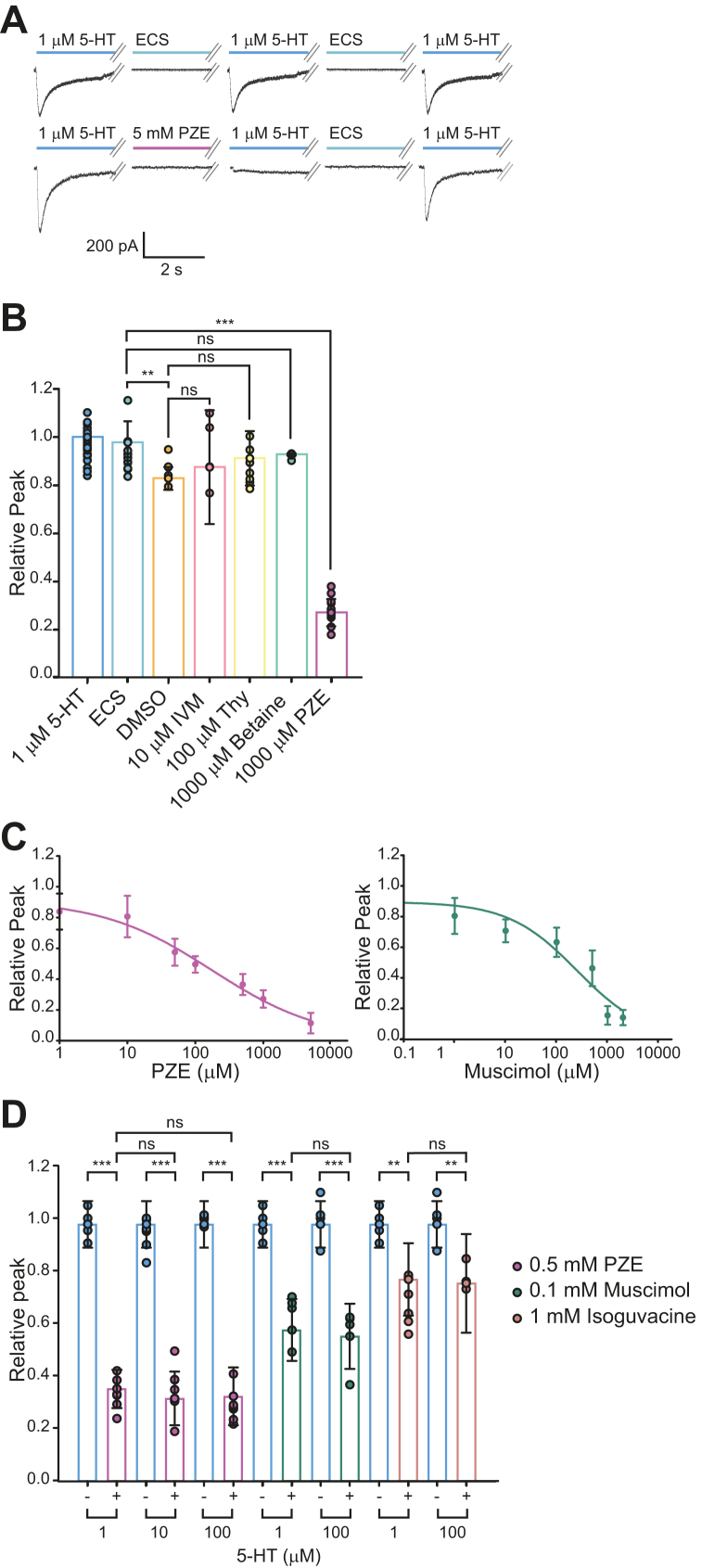
**Modulation of MOD-1 currents by preincubation with different compounds**. *A*, illustrative example of the protocol used to determine the effects of drug preincubation on 5-HT-elicited currents (preincubation protocol). Holding potential: -50 mV. 3.5 s-pulse of 1 μM 5-HT was applied before and after preincubation during 30 s with ECS (control) or the drug under study. Currents were recorded after 30 s wash with ECS to confirm recovery. This protocol was repeated three times in each cell. *B*, plot showing the effect of different ligands at the indicated concentrations on 5-HT elicited currents. The individual data points for each condition are shown with the colored bar indicating the mean ± SD. Student’s *t* test, *p*-values for IVM: ivermectin (10 μM, n = 11, *p* = 0.675); Thy: thymol (100 μM, n = 3, *p* = 0.183); betaine (1 mM, n = 8, *p* = 0.48); PZE: piperazine (1 mM, n = 12). ∗∗ *p* < 0.01, ∗∗∗ *p* < 0.001 and ns: not statistically significant. *C*, concentration–response curve showing the inhibition of MOD-1 currents by PZE or muscimol. The curve was fitted by a logistic function using Sigma Plot. Each point corresponds to the normalized current at a given concentration from at least three different experiments. *D*, effects of 0.5 mM PZE (n = 6, *p* = 1), 0.1 mM muscimol (n = 6, *p* = 1), and 1 mM isoguvacine (n = 6, *p* = 1) on the peak currents elicited by different concentrations of 5-HT. The individual data points for each condition are shown with the colored bar indicating the mean ± SD. Comparisons were performed by ANOVA with Bonferroni *t* test. ∗∗ *p* < 0.01, ∗∗∗ *p* < 0.001 and ns: not statistically significant. ECS, extracellular solution; 5-HT, serotonin.

For evaluating modulation of MOD-1, we followed a preincubation protocol in which the cell was exposed to the compound before the application of 5-HT. A pulse of ECS containing 1 μM 5-HT (control current) was applied to the cell, the cell was then exposed during 30 to 120 s to ECS containing the drug under study, and, finally, a second pulse of ECS containing 1 μM 5-HT was applied (treated current). The changes in net charge and amplitude of the treated current with respect to the control one were averaged among different cells (Fig. 3*A*).

Thy at 5 μM (n = 3), 50 μM (n = 3), and 100 μM (n = 3); betaine at 1 mM (n = 8), 5 mM (n = 3), and 10 mM (n = 3); and IVM at 10 μM (n = 11) did not have any significant effect on 5-HT elicited currents (Fig. 3*B*). Interestingly, pre-exposure of the cell to 1 mM PZE led to a profound decrease of the peak current (69.2 ± 6%, *p* < 0.001) and of the net charge (66.3 ± 9%, *p* < 0.001, n = 12) (Fig. 3*B*).

To further characterize the action of PZE as a new inhibitor of MOD-1, we constructed concentration–response curves to determine the IC_50_ (half maximal inhibitory concentration) for currents activated by 1 μM 5-HT and preincubated for 30 s with PZE (Fig. 3*C*). We found that PZE inhibits MOD-1 currents with an IC_50_ of 113 ± 29 μM.

We determined if PZE also affects MOD-1 currents when applied together with 5-HT (co-application protocol). In each cell, we applied three pulses of 1 μM 5-HT-containing ECS and then three pulses of ECS containing 1 μM 5-HT and 1 mM PZE. PZE produced neither statistically significant changes in the peak currents (0.91 ± 0.09, *p* = 0.617) nor in the net charge (0.93 ± 0.19, *p* = 0.669, n = 5). Thus, in contrast to the important effects observed with preincubation, co-application of PZE with 5-HT does not inhibit MOD-1 currents.

Since PZE is a GABAergic ligand and MOD-1 shows ∼28% identity with Cel-UNC-49 (γ-aminobutyric acid type A receptor [GABA_A_]) receptors, we also tested, by using the preincubation protocol, the effects of muscimol—a psychoactive isoxazole from *Amanita muscaria* and related mushrooms—and isoguvacine, which are selective GABA_A_ agonists (35). We found that both drugs were not able to activate MOD-1 but produced inhibition of MOD-1 currents elicited by 5-HT. At 1 mM, isoguvacine reduced the peak currents 23 ± 14% (n = 10) and muscimol, 85 ± 6% (n = 5). Isoguvacine appeared to be a very weak antagonist since the reduction of the peak current remained at about 30% at concentrations higher than 0.6 mM.

To further characterize the action of muscimol as a new inhibitor of MOD-1, we constructed concentration–response curves to determine the IC_50_ value for currents activated by 1 μM 5-HT and preincubated for 120 s with muscimol (Fig. 3*C*). We found that muscimol inhibits MOD-1 currents with an IC_50_ of 249 ± 160 μM, indicating lower potency than PZE.

The inhibition of 5-HT currents by the GABAergic ligands could be due to either competitive or noncompetitive antagonism. The fact that PZE exerted greater reduction of currents if it was preincubated and not co-applied with 5-HT suggests that the inhibition is not competitive, thus revealing a new drug target for this classical antiparasitic agent. To further confirm the type of antagonism, we performed experiments in which the concentration of PZE during preincubation remained constant, but the 5-HT concentration was increased 10 and 100 times. The rationale for this experiment was that if PZE were competing with 5-HT, the increase in 5-HT concentration would reduce its effect. Figure 3*D* shows that the decrease in the peak current due to the preincubation with 0.5 mM PZE was independent of the agonist concentration (1, 10 or 100 μM 5-HT), suggesting noncompetitive inhibition. As described for PZE, the percentage of current reduction did not change significantly with 5-HT concentration (1 or 100 μM) for 1 mM isoguvacine or 0.1 mM muscimol, indicating a similar mechanism of noncompetitive antagonism (Fig. 3*D*).

### *In vivo* effects involving MOD-1 modulation

To relate the molecular effects of MOD-1 ligands to behavioral effects at the organism level, we performed locomotor activity assays in *C. elegans*. All assays were carried out in parallel with control experiments, and the main effects were confirmed by visual inspection using the thrashing assay method (Fig. S1).

**Figure 4:**
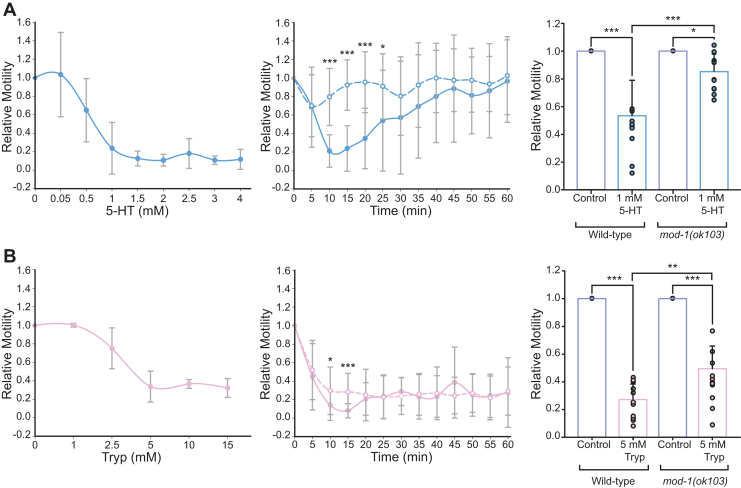
**Behavioral effects of ligands acting through MOD-1**. Synchronized young adult wildtype worms were placed on 96-microwell plates (about 50 worms per well) containing 5-HT (*A*) or Tryp (*B*), and motility was recorded using the WMicroTracker in 5-min intervals during 120 min, first 60 min are shown. *A*, reduction of worm motility as a function of 5-HT concentration and time. *Left*: Concentration–response curve was constructed by measuring the reduction of motility at 15 min of exposure to the indicated 5-HT concentrations. The representative experiment shown corresponds to the mean ± SD of 12 wells for each condition (with about 50 worms per well) run in *parallel*. *Middle*: Motility was recorded as a function of time for synchronized wildtype (*filled*) and *mod-1(ok103)* (*dashed line*) worms in the absence (time 0, 100% motility) or presence of 1 mM 5-HT. The curve corresponds to the average of 11 independent experiments, each with 12 wells per condition run in parallel. *Right*: Bar chart showing the reduction of worm motility by 1 mM 5-HT at 15 min of exposure for wildtype and *mod-1(ok103)* worms (n = 11 independent experiments). The results are shown as mean ± SD. About 50 worms were used per well and a minimum of 12 wells for each condition in each independent experiment. Comparisons were performed by ANOVA with Bonferroni *t* test. *p*-values were ∗∗∗*p* < 0.001 and ∗*p* = 0.026. *B*, reduction of worm motility as a function of Tryp concentration and time. Experiments were performed as described for 5-HT. The representative concentration–response curve corresponds to the mean ± SD of 12 wells for each condition with about 50 worms per well. The *right plots* show the individual data points for each experiment with the colored bar indicating the mean ± SD. Each individual point corresponds to one of the 13 independent experiments, each from 12 wells (50 worms per well) per condition. Comparisons were performed by ANOVA with Bonferroni *t* test. *p*-values for n = 13 independent experiments were ∗∗∗*p* < 0.001; ∗∗*p* = 0.006. 5-HT, serotonin; Tryp, tryptamine.

We first explored how 5-HT affects the whole organism by evaluating the response of young adult wildtype worms to 5-HT exposure as a function of time and concentration (0.05–4 mM in water) (Fig. 4*A*). We found that 1 mM 5-HT in water produced a rapid and reversible decrease of motility of wildtype worms. The reduction of motility reached its maximal level, which was between 50 and 80%, after 15 min of exposure to the drug (Fig. 4*B*, *p* < 0.001). However, wildtype worms recovered from this quiescent state gradually after 20 min of 5-HT exposure in most of the assays. After 60 min, worm motility was close to its basal levels (⁓90% recovery) even in the continued presence of 1 mM 5-HT (Fig. 4*A*). In contrast, we observed that at 5-HT concentrations higher than 1.5 mM (n = 5), worms did not recover from the paralysis at least during the first 120 min.

The *mod-1(ok103)* mutant strain, which lacks MOD-1, was partially resistant to 1 mM 5-HT since motility was reduced only about 10 to 20%, thus indicating that MOD-1 is a target for 5-HT motility inhibition (Fig. 4*A*). The difference of the inhibition between wildtype and mutant worms after 15-min exposure to 1 mM 5-HT was statistically significant (*p* < 0.001, Fig. 4*A*).

The mutant worms also recovered with time, indicating that the reversibility of 5-HT effect was not dependent on MOD-1 alone. Furthermore, at higher 5-HT concentrations, (>2 mM) MOD-1 mutant worms were completely paralyzed as wildtype worms.

We also explored how Tryp affects young adult *C. elegans* motility. A clear reduction of worm motility was observed after exposure to 5 mM Tryp. The major effect was achieved after 15 min, at which a statistically significant reduction of worm motility occurred (>70%, *p* < 0.001) (Fig. 4*B*). The decrease of activity in the continued presence of 5 mM Tryp was more pronounced for wildtype than for *mod-1(ok103)* worms (⁓72% and ⁓50%, respectively; *p* = 0.006). Thus, as shown for 5-HT, MOD-1 is partially involved in the Tryp-paralyzing effect. Differently to 5-HT, worms did not recover to basal activity values during 120 min-exposure (Fig. 4, *A* and *B*).

**Figure 5:**
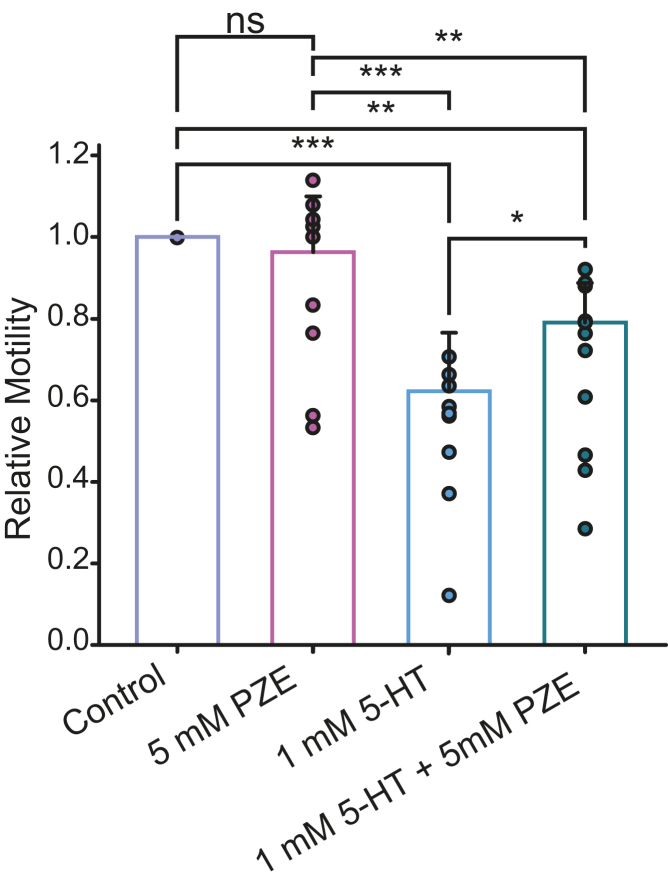
**Pharmacological effects of PZE acting through MOD-1**. Plot showing the change in motility after 15-min exposure to the indicated drugs in wildtype worms. Control corresponds to worms’ basal movement in the absence of drugs. The individual data points for each condition are shown with the colored bar indicating the mean ± SD for nine independent assays. Comparisons were performed by ANOVA with Bonferroni *t* test. *p*-values from *left to right* were: ∗∗∗*p* < 0.001; ∗∗*p* = 0.002; ∗∗∗*p* < 0.001; ∗∗*p* = 0.009; ∗*p* = 0.027 and ns: not statistically significant. 5-HT, serotonin; PZE, piperazine.

We sought to evaluate if the inhibitory action of PZE on MOD-1 receptor affects the worm response to 5-HT. The first step was to dissect this effect from its agonistic action on muscle UNC-49 (GABA_A_) receptor that leads to flaccid paralysis. Thus, we tested PZE concentrations at which flaccid paralysis did not occur as revealed by both automatic activity assays and thrashing assays. As shown in Figures 5 and S1, no changes in motility were observed at 5 mM PZE after 15 min exposure, whereas at 10 mM PZE, the paralysis was evident. Previous reports have shown that high PZE concentrations were necessary to produce an anthelmintic effect (27).

The combination of 1 mM 5-HT, which activates MOD-1, with 5 mM PZE, which inhibits MOD-1, produced smaller motility inhibition than that produced by 1 mM 5-HT alone. These results indicate that PZE attenuates the paralysis induced by exogenous 5-HT (Fig. 5). Thus, these experiments confirm that PZE inhibits MOD-1 in the whole organism.

## Discussion

MOD-1 receptor is a pentameric serotonin-gated chloride channel that is unique to nematodes, a feature that makes it a promising anthelmintic target. It shows ⁓80% similarity to *H. contortus* MOD-1 (Hco-MOD-1; (17)) and ⁓46% to the plant parasitic nematode *G. pallida* MOD-1 (Gpa-MOD-1; (18)). The high conservation of MOD-1 within the phylum Nematoda reinforces the use of *C. elegans* as a model in anthelmintic drug discovery. Here we decipher novel aspects of MOD-1 molecular function and identify ligands that acting by different mechanisms may provide leads for new anthelmintic development. The identification of MOD-1 as a new target of the classical antiparasitic PZE shows that it behaves as a multitarget drug. Multitarget drugs have recently attracted much attention as promising tools to fight against complex diseases and against those linked to drug resistance issues, such as parasitic diseases (36).

The establishment of a novel drug target requires knowledge of its pharmacology and function at the molecular level. With this in mind, we expressed MOD-1 in mammalian cells, characterized its activation properties, and performed a pharmacological screening for identifying MOD-1 ligands using whole-cell patch clamp recordings. We found that MOD-1 expresses well and functions as a homomeric receptor in BOSC-23 cells as described before (10). The macroscopic current recordings show that MOD-1 responses are rapidly elicited by 5-HT, similarly to vertebrate 5-HT_3_A receptors, decay in the presence of the agonist due to desensitization and recover relatively rapid from desensitization in the absence of agonist. Currents are of similar amplitudes at positive and negative membrane potentials, indicating no significant rectification.

5-HT_3_ and MOD-1 have evolved from a common ancestral gene and respond to 5-HT, but MOD-1 is anionic and absent in vertebrates (13). The EC_50_ determined for MOD-1 from the peak currents as a function 5-HT concentration is in the same order as that of vertebrate 5-HT_3_A receptors (21). However, MOD-1 responds very differently to 5-HT_3_A partial agonists. In this regard, Tryp is a significantly more efficacious and potent agonist of MOD-1 than of 5-HT_3_A (21). In contrast, 2-Me-5HT, which efficaciously activates 5-HT_3_A receptors (21), cannot activate MOD-1, thus revealing different agonist selectivity between these two 5-HT-activated Cys-loop receptors. Thus, although they share 5-HT as their endogenous neurotransmitter, they differ in how it interacts at the binding site (37).

In Cys-loop receptors, the binding site is located at interfaces between adjacent subunits, and it is composed of residues from loops A, B, and C on the principal (+) subunit and loops D, E, and F from the complementary (−) subunit (38, 39). Conserved aromatic residues of different loops have been shown to be important for agonist recognition and activation in all the family. In the agonist binding site region, 5-HT_3_ and MOD-1 are highly homologous (37). However, there are differences among key aromatic residues. The conserved tryptophane residue in loop B of 5-HT_3_, which makes a cation-π interaction with 5-HT important for activation (21, 37, 39, 40, 41), is replaced by a tyrosine in MOD-1. Also, a conserved 5-HT_3_ tyrosine in loop C is a tryptophane in MOD-1. It has been therefore suggested that the critical cation-π binding interaction from loop B in 5-HT_3_ moved to loop C site in MOD-1 (37). Thus, although MOD-1 and 5-HT_3_ receptors have been precisely designed to bind 5-HT, they respond differently to different serotoninergic ligands. Recent high-resolution cryo-electron microscopy structures of full-length 5-HT_3_A in complex with 5-HT or different setrons, which are competitive antagonists of 5-HT_3_ receptors, combined with molecular dynamics simulations revealed distinct interaction fingerprints for the ligands at the binding pocket that may underlie their diverse affinities (39, 42). Thus, structural studies on MOD-1 may be required to decipher the basis of drug selectivity. Indeed, high concentrations of granisetron and ondansetron, both of which are potent antagonists of 5-HT_3_A, did not affect MOD-1 macroscopic responses to 5-HT (10). Also, Thy, an allosteric agonist of 5-HT_3_A receptors (22), cannot activate MOD-1, thus indicating different mechanisms or sites for ago-PAMs between both receptors.

Our behavioral study shows that the exposure to a low 5-HT concentration produces a transient paralysis from which worms recover in the continuous presence of 5-HT within 60 min in most of the assays. This recovery—to our knowledge—has not been reported in previous studies (10, 18, 19), probably because of the high 5-HT concentrations used in the assays. Indeed, in our assays at 5-HT concentrations higher than 1 mM, worm motility remains low after 120 min. Regarding adaptative responses to exogenous 5-HT, it has been described that worms exposed to high 5-HT concentrations recovered after being transferred to a 5-HT-free environment (43) and that worms can adapt to the egg laying stimulation induced by 5-HT after a long-term exposure to high 5-HT concentrations (44).

The recovery of worm motility observed in the continuous presence of 1 mM 5-HT may be due to reuptake of 5-HT to neuronal terminals by the serotonin reuptake transporter MOD-5, 5-HT degradation during the assay—although freshly prepared 5-HT solutions are used in each assay—or receptor desensitization and/or stress adaptation to 5-HT ambient. This novel effect should be considered in future 5-HT signaling studies.

The inhibition of motility produced by 5-HT is less profound in mutants lacking MOD-1 than in wildtype worms, indicating partial contribution of MOD-1 to the 5-HT paralysis, which, in turn, is consistent with the presence of other 5-HT receptor types.

It has been recently described that Tryp activates LGC-50 (12), and this is the first report, to our knowledge, showing that Tryp is an activator of *C. elegans* MOD-1. Our electrophysiological recordings show that Tryp is an efficacious agonist of MOD-1 (α ⁓80%), and our behavioral studies reveal that it reduces worm motility. Moreover, the comparison between wildtype and MOD-1 mutant worms shows the partial contribution of MOD-1 to this paralysis. In contrast to our observations with 5-HT, recovery of motility does not occur with Tryp, which may be due to different reuptake or degradation properties of the two ligands. Most of the synthesis pathways of monoamine neurotransmitters are multistep processes. In vertebrates, Tryp is synthetized from tryptophane by aromatic amino acid decarboxylase enzyme and acts as a trace amine. Although there are several uncharacterized enzymes in *C. elegans* genome that could be involved in the generation of Tryp, it has not been detected in *C. elegans* to date (45). Thus, the possible absence of endogenous Tryp may be one of the factors for the absence of recovery of worms since they may lack specific mechanisms for degradation or reuptake; therefore, Tryp may behave as a xenobiotic.

Our electrophysiological and behavioral studies of Tryp effects on MOD-1 that allow the evaluation of this compound from the molecular level to the organism level reveal a marked anthelmintic effectivity. Hence, Tryp-derived agents may be promising compounds for further antiparasitic drug research.

A previous characterization of MOD-1 showed that it is not activated by the neurotransmitters glycine, ACh, GABA, glutamate, and histamine (10). As several anthelmintic agents act as agonists of Cys-loop receptors, we tested their potential to activate MOD-1. Our whole-cell recordings show that PZE, betaine, LEV, and IVM do not act as agonists of MOD-1, thus increasing our knowledge of MOD-1 pharmacology.

In our search for MOD-1 modulators, we first tested modulators of Cys-loop receptors. In this regard, IVM has been shown to have a broad spectrum of action, being an allosteric agonist or positive and negative allosteric modulator at different Cys-loop receptors (5, 46). Here we show that it is does not exert an important modulation of MOD-1 currents. Interestingly, we reveal that the GABAergic agonists, PZE, muscimol, and isoguvacine, act as inhibitors of MOD-1.

Among the tested ligands, PZE is the most potent inhibitor. Our results showing that the extent of inhibition of peak currents by the GABAergic ligand does not depend on 5-HT concentration and that the preincubation with the drug exerts a more profound effect that its co-application suggest that the inhibition is due to a negative allosteric modulation. Previously, it was shown that mianserin and methiothepin act as noncompetitive antagonists of MOD-1 (10). Thus, by identifying novel MOD-1 modulators, our electrophysiological studies open doors for the rational design of future anthelmintic lead compounds.

The binding sites for GABAergic ligands as modulators of MOD-1 remain unknown. Cys-loop receptors contain an intersubunit transmembrane cavity at which several ligands, including IVM, may bind with different locations and orientations (38). Future structural studies may enlighten our understanding of MOD-1 modulatory sites.

By using motility assays that measure worm responses to exogenous 5-HT, we reveal that the PZE inhibition of MOD-1 is translated into *in vivo* effects. At the behavioral level, it is even more potent for counteracting the effects of exogenous 5-HT than for producing paralysis through the activation of GABA receptors. Thus, MOD-1 is a novel target for PZE.

We therefore propose that PZE-derived ligands could be explored as anthelmintic drugs and that the inhibition of MOD-1 by PZE may be a novel mechanism that acts synergically to its classical anthelmintic action as agonist of GABA receptors. Multitarget drug treatment approach is important when the monotherapy seems to be failing, particularly in diseases associated with the increasing incidences of drug resistance. Due to the high conservation of MOD-1 receptors within the phylum Nematoda, our findings could be extrapolated to parasitic species, thus revealing a new drug target for this antiparasitic agent. There is little information regarding MOD-1 as an antiparasitic drug target, and its function is still poorly known. However, an antiparasitic mechanism involving MOD-1 has been proposed for plant parasitic nematodes. Methiothepin acting as an antagonist of MOD-1 or SER-7 was shown to inhibit *G*. *pallida* stylet thrusting and root invasion (18), highlighting the potential of antagonists of MOD-1 as antiparasitic drugs.

In conclusion, our study from the molecular level to the organism level enhances our knowledge of Cys-loop receptors, provides support for MOD-1 as a promising target for anthelmintic therapy, and identifies lead compounds that by activating or inhibiting MOD-1 may be explored as antiparasitic drugs.

## Experimental procedures

### *C. elegans* strains and culture

Nematode strains used were as follows: N2: Bristol wild type; PD4251: *ccIs4251;dpy-20(e1282)*; and MT9668: *mod-1(ok103)*. All strains were obtained from the *Caenorhabditis* Genetic Center, supported by the 10.13039/100000002National Institutes of Health—Office of Research Infrastructure Programs (P40 OD010440). Nematodes were maintained at 18 to 25 °C using freshly prepared Nematode Growth Medium petri dishes spread with *Escherichia coli* (OP50) as a source of food as described before (25, 28).

### Locomotion assays

Motility assays were performed with WMicroTracker (Phylumtech), which is a LED-based assay system that allows drug effects on nematode motility to be recorded over time (47, 48). All behavioral assays were done at room temperature (21–23 °C) with young adult hermaphrodite worms from synchronized plates.

Prior to the experiment, worms were transferred from Nematode Growth Medium agar plates into a 15 ml conical tube containing water, allowed to sink to the bottom and washed three times with water. Then, animals were transferred to flat bottomed 96-well microplates at an average of 50 worms per well in water. The experiments were carried out in water since less 5-HT is required than in salt-containing media (20). Worms’ basal movement was measured for 30 min to normalize the movement activity for each well at the beginning of the assay (basal, 100% activity). Then, drugs were added to a final volume of 100 μl per well. All drugs were tested with a minimum of 12 replicates per plate. For comparison among different drug concentrations or worm strains, the assays were performed in parallel. Motility values for different experimental groups were analyzed in 5-min time bins during 60 to 120 min. Each condition was evaluated in at least five independent experiments with different synchronized worm batches and always in parallel with the respective control (25, 28). 5-HT and Tryp solutions were freshly prepared for each assay.

The thrashing assay method was used to visually confirm the results obtained with WMicroTracker. Synchronized worms were placed in individual wells containing 100 μl of H_2_O with or without drug at room temperature in a 96-well microtiter plate. After 15 min, the number of thrashes (bends of the body from one side to the other) were counted during 1 min. All the experiments were carried out at least three independent times (n > 15 worms were analyzed per condition in each experiment) (27, 49).

### Heterologous cell expression of MOD-1 receptors

MOD-1 was transiently expressed in BOSC-23 cells, which are modified HEK 293T cells (50). The cDNA encoding the *C. elegans* MOD-1 was generously provided by Dr Horvitz (The Horvitz Lab, Massachusetts Institute of Technology) and was subcloned into the pcDNA3.1 vector. Cells were transfected by calcium phosphate precipitation with MOD-1 and GFP cDNAs (total 4 μg/35 mm dish) essentially as described before (22, 51, 52). All transfections were carried out for about 8 to 12 h in Dulbecco's modified Eagle's medium with 10% fetal bovine serum and were terminated by exchanging the medium. Cells were used for whole-cell recordings 2 to 3 days after transfection (52).

### Whole-cell recordings from BOSC-23 cells

Macroscopic currents were recorded in the whole-cell configuration as described previously (52). The pipette was filled with intracellular solution containing 134 mM KCl, 5 mM EGTA, 1 mM MgCl_2_, and 10 mM Hepes (pH 7.3). The ECS contained 150 mM NaCl, 1.8 mM CaCl_2_, 1 mM MgCl_2_, and 10 mM Hepes (pH 7.4). After whole-cell formation, ECS containing the agonist was rapidly applied using a three-tube perfusion system with elevated solution reservoirs for gravity-driven flow and switching valves controlled by a VC3 controller (ALA Scientific). The solution exchange time was estimated by the open pipette method, which consists in applying a pulse of 50% diluted ECS to an open patch pipette to produce a sudden change in the current measured by the patch-clamp amplifier (53). Proper adjustment of the electrode position allowed a current jump in our system to be between 0.1 and 1 ms (28). The compounds were dissolved in ECS from water or DMSO stock solutions. The final concentration of DMSO was lower than 0.02% for electrophysiological assays.

To study the modulatory actions of different compounds, responses were evaluated following co-application or preincubation protocols. For the co-application protocol, three pulses of 5-HT-containing ECS were applied in each cell (control current) and then three pulses of ECS containing 5-HT and the tested compound. For preincubation protocols, a pulse of ECS containing 5-HT (control current) was first applied to the cell held at -50 mV, and the cell was incubated during 30 to 120 s with ECS containing the drug under study before a second pulse of ECS containing 5-HT was applied (treated). At the end of each protocol, control currents were again recorded to assess recovery of the original peak current. Unless specified, the duration of the 5-HT pulse was 3.5 s. A wash with ECS alone (30–120 s) allowed total recovery of control currents. The changes in net charge and amplitude of the second (treated) current with respect to the control one were determined for each cell and averaged among different cells.

Currents were filtered at 5 kHz and digitized at 20 kHz using an Axopatch 200B patch-clamp amplifier (Molecular Devices) and acquired using WinWCP software (Strathclyde Electrophysiology Software, University of Strathclyde, Glasgow, UK). The recordings were analyzed using the ClampFit software (Molecular Devices). Currents were fitted by a double exponential function according to the equation:

$$I\;\left(t\right)\;=\;I_{\mathrm{fast}}\left[\exp\left({\mathrm{t/\tau}}_{\mathrm{fast}}\right)\right]\;+\;I_{\mathrm{slow}}\left[\exp\left({\mathrm{t/\tau}}_{\mathrm{slow}}\right)\right]\;+\;I_{\infty }$$

in which t is time, I is the peak current, I_∞_ is the steady state current value, and τ are the decay time constants. Net charge was calculated by current integration (54, 55). The rise time corresponds to the time taken by the current to increase from 10% to 90% of its maximal value (t_10-90%_).

For constructing the current–voltage (I/V) curve, we determined the peak current at different membrane potentials. The peak current was related to that obtained at a holding potential of −100 mV for each cell and was averaged for three different independent cells and experiments. The data were fitted by linear regression. The chloride concentrations were [Cl^-^]_out_ = 155.6 mM and [Cl^-^]_in_ = 136 mM, corresponding to the ECS and intracellular solution, respectively. Under our experimental conditions, the Nernst equation predicts an equilibrium potential for chloride of −3.4 mV.

Recovery from desensitization was determined by using a twin pulse procedure as described before (56). Whole-cell currents at -50 mV were elicited by a 3.5 s step pulse of 100 μM 5-HT that achieved full desensitization. Then, a pulse of free-agonist ECS of variable durations (200-ms intervals) was applied, and finally, a second pulse of agonist was applied. Fractional recovery was determined as the ratio between the peak current elicited by the second pulse respect to the first one for each cell. Values of fractional recoveries were averaged for different cells and plotted against the corresponding time of exposure to the agonist-free pulse. The points were fitted using SigmaPlot 12.0 (Systat Software Inc) by the exponential rise equation:

$$f\;\left(t\right)=a\left(1-\exp\left(-t/\tau_{r}\right)\right)$$

in which *t* is the interpulse interval, and τ_r_ is the recovery time constant.

### Data and statistical analysis

Experimental data are shown as mean ± SD. Statistical comparisons were done using two-tailed Student’s *t* test for pairwise comparisons or one-way ANOVA followed by Bonferroni’s post hoc tests for multiple comparisons. Statistically significance was established at *p* values < 0.05 (∗*p* < 0.05, ∗∗*p* < 0.01, ∗∗∗*p* < 0.001). All the tests were performed with SigmaPlot 12.0 (Systat Software, Inc). Concentration–response curves were fitted by a logistic function using Sigmaplot 12.0, from which EC_50_ (half maximal effective concentration) or IC_50_ (half maximal inhibitory concentration) values were obtained and expressed as mean ± SE.

## Data availability

All data are contained within the manuscript.

## Supporting information

This article contains supporting information.

## Conflict of interest

The authors declare that they have no conflicts of interest with the contents of this article.
