# Supplementary material for: The nematode serotonin-gated chloride channel MOD-1: A novel target for anthelmintic therapy
Source: J Biol Chem. 2022 Aug 9;298(9):102356. doi: 10.1016/j.jbc.2022.102356 (PMC9471462; doi:10.1016/j.jbc.2022.102356)
Supplement: Supporting information [file mmc1.pdf]

The nematode serotonin-gated chloride channel MOD-1: A novel target for anthelmintic therapy

Noelia Rodriguez Araujo, Guillermina Hernando, Jeremías Corradi and Cecilia Bouzat

Instituto de Investigaciones Bioquímicas de Bahía Blanca, Departamento de Biología, Bioquímica y Farmacia, Universidad Nacional del Sur-Consejo Nacional de Investigaciones Científicas y Técnicas (CONICET), Bahía Blanca, Argentina.

Supporting information

Figure S1. Effects of 5-HT, tryptamine and piperazine on nematode swimming measured as thrashes per minute for wild type worms.

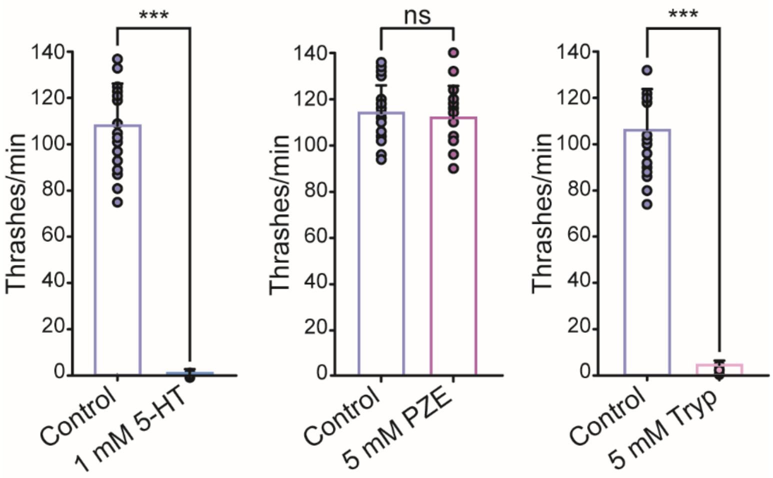

Synchronized worms were placed in individual wells containing 100 µl of H_2O with or without drug at room temperature in a 96-well microtiter plate. After 15 min, the number of thrashes (bends of the body from one side to the other) were counted during 1 min. All the experiments were carried out at least three independent times (n > 15 worms were analyzed per condition in each experiment). The individual points for each condition are shown with the colored bar indicating the mean ± SD. Student's t-test. p-values were: ***p<0.001; ns: not statistically significant (p=0.723).
